# The foggy effect of egocentric distance in a nonverbal paradigm

**DOI:** 10.1038/s41598-021-93380-9

**Published:** 2021-07-13

**Authors:** Bo Dong, Airui Chen, Yuting Zhang, Yangyang Zhang, Ming Zhang, Tianyang Zhang

**Affiliations:** 1 Department of Psychology, Suzhou University of Science and Technology, 99 Xuefu Road, Huqiu District, Suzhou, 215009 Jiangsu China; 2 School of Psychology, Shaanxi Normal University, 199, South Chang’an Road, Yanta District, Xi’an, 710062 China; 3 Department of Psychology, Soochow University, 50 Donghuan Road, Gusu District, Suzhou, 215021 Jiangsu China; 4 School of Public Health, Soochow University, 199 Renai Road, Industry Park, Suzhou, 215123 Jiangsu China

**Keywords:** Psychology, Psychology and behaviour

## Abstract

Inaccurate egocentric distance and speed perception are two main explanations for the high accident rate associated with driving in foggy weather. The effect of foggy weather on speed has been well studied. However, its effect on egocentric distance perception is poorly understood. The paradigm for measuring perceived egocentric distance in previous studies was verbal estimation instead of a nonverbal paradigm. In the current research, a nonverbal paradigm, the visual matching task, was used. Our results from the nonverbal task revealed a robust foggy effect on egocentric distance. Observers overestimated the egocentric distance in foggy weather compared to in clear weather. The higher the concentration of fog, the more serious the overestimation. This effect of fog on egocentric distance was not limited to a certain distance range but was maintained in action space and vista space. Our findings confirm the foggy effect with a nonverbal paradigm and reveal that people may perceive egocentric distance more "accurately" in foggy weather than when it is measured with a verbal estimation task.

## Introduction

The accident rate is much higher when driving in foggy weather than in clear weather. Two kinds of dangerous driving behaviors occur more frequently in foggy weather: smaller headways during car following and excessive driving speed during lane keeping. The misestimation of speed and the overestimation of egocentric distance are typically thought to be the main perceptual mechanisms for these risky driving behaviors^1^.

The effect of foggy weather on speed has been well studied. Recent studies have suggested that the direction of the misestimation is determined by the polarity of contrast between the peripheral and central areas of the visual field^2^. When visibility is better in the peripheral than in the central visual field, as is the case in fog, speed is overestimated^2–5^. Uniform contrast reduction decreases the perceived speed of driving^6–8^, consistent with the Thompson effect^9^. The Thompson effect is not attenuated when distance cues are enhanced by the addition of binocular depth cues^10^. Conclusions about speed perception in foggy weather have been demonstrated through verbal estimation^7,11^ as well as nonverbal paradigms, such as speed matching^2,6,8^, motion discrimination^8,12,13^, driving at a certain speed^2,6^, interceptive actions^14^, and even speed choice in lane-keeping tasks^15^.

In contrast to the established effect of foggy weather on speed, the overestimation of the egocentric distance of vehicles in front is poorly understood. To the best of our knowledge, Ross was the first to show that participants perceived a larger egocentric distance in foggy weather than in clear weather^16,17^. Cavallo and her colleagues subsequently conducted a series of systematic experiments in a fog chamber to confirm this overestimation pattern^1,4,18–20^. The perceived distance in foggy weather were 20–30% higher on average than those in clear weather during the day because of the reduction in depth cues (e.g., luminance contrast, blurring, aerial perspective)^18,19^. The degree of overestimation can reach 55–60% at night as depth cues weaken or disappear (e.g., the contour of objects, linear perspective, luminance)^4,18,19^. It is important to note that the method for measuring the perceived distance in these studies was verbal estimation: participants were asked to report the perceived distance orally in meters/feet/inches. Classical nonverbal paradigms, such as visual matching, blind walking, blindfolded throwing, blind rope pulling, and triangulation by pointing, have not been used previously in distance perception studies.

It is well known that the popularity and accuracy of nonverbal paradigms are better than those of verbal estimation in measuring distance perception^21–24^. For example, we can easily pick up a cup on a table. However, it is difficult to orally report exactly how far the cup is from us. Whereas perceived egocentric distance is proportional to physical distance in both verbal estimation and nonverbal paradigms, verbal estimation is often accompanied with more underestimation (linear functions with slopes near 0.7–0.8) than nonverbal paradigms (slopes near 1.0)^22,23,25,26^. The intrasubject reliability is better in nonverbal paradigms than in verbal estimation paradigms^27^. Unlike ordinary psychophysical studies, studies of distance perception in foggy weather usually involve targets more than 30 m away. In this context, blind walking, blindfolded throwing, and other action-based paradigms are not applicable well. It is difficult for a person to walk more than 30 m with his or her eyes closed or to throw an object 30 m away. Thus, a visual matching task is more appropriate in this case.

Considering these issues, we employed a visual matching task in combination with a virtual reality (VR) technique to explore egocentric distance perception in foggy weather. The VR technique was used to generate an environment with different fog concentrations. The visual matching task was used to assess perceived egocentric distance in foggy and clear weather. Observers evaluated the egocentric distance of a test target (i.e., a red ball) on the ground and then adjusted the egocentric distance of a matching target ball (i.e., a basketball) until they felt the two balls were at the same egocentric distance. In this way, participants reported their perceived egocentric distance nonverbally. We were mainly interested in one critical issue: how foggy weather modifies observers’ egocentric distance perception in this nonverbal paradigm.

## Experiment 1

In a virtual environment, a visual matching task was used to test the effect of fog concentration on distance perception. Experiment 1 was a two-factor repeated-measures design. The first independent variable was the fog concentration. The concentrations of fog in the VR environment were 0, 0.02, 0.04, and 0.06. A higher value indicates a higher concentration of fog. The second independent variable is the physical distance of the target. The physical distance between observers and the target was 5 m, 10 m, 15 m, 30 m, or 50 m. The goal of the current research was to examine whether the fog concentration generated by the virtual visual system could modulate egocentric distance perception in the action space (> 2–3 m and < 30 m) and vista space (> 30 m).

### Methods

#### Participants

Twenty participants (19 women, one man) aged 18–23 years old were recruited. The participants were naïve to the purpose of our study. All of them had normal or corrected-to-normal vision and gave informed consent prior to the experiment. They received payment after the experiment. This research was approved by the local ethics committee of Suzhou University of Science and Technology and was conducted in accordance with the guidelines of the Declaration of Helsinki.

#### Apparatus and stimuli

An Alienware Area-51 Thread with the GTX1080 graphics card manipulated the virtual system (HTC Vive), which included a head-mounted display (HMD) with two screens (90 fps, 1080 × 1200 pixel resolution, 110° field of view), lighthouses, gyroscopes, sensors for tracking participants’ movements and location (0.1° precision), and two hand controllers. The binocular viewing resolution was 2160 × 1200 pixels. The experimental database and stimuli were constructed using World Vizard 6 software.

**Figure 1:**
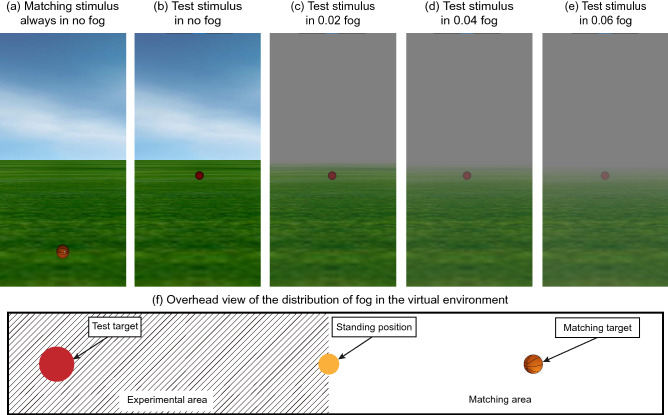
The virtual environment in experiment 1. Matching stimulus is a basketball, which is always in the no fog environment (**a**). Test target is a red ball, which is under the no-fog (i.e., clear weather) (**b**), the 0.02 fog-concentration (**c**), the 0.04 fog-concentration (**d**), and the 0.06 fog-concentration (**e**) conditions. To illustrate the experimental scene, we draw the overhead view of distribution of fog in the virtual environment (**f**).

The virtual environment consisted of ground, sky, targets, and white clouds (see Fig. 1a–e). A texture gradient was applied to the ground. The entire 360° world was visible by looking around. The horizon was where the sky and grass met, and its height was set to the participant's eye height. The three-dimensional coordinates of the yellow landmark where participants stood were set at 0, 0, 0 (x, y, z). The test target was randomly selected from five balls. To prevent participants from evaluating the distance based on the size of the ball, all balls were projected to the participants’ retinas at the same size (1° visual angle). The diameters of the five balls were 8.73 cm, 17.46 cm, 26.18 cm, 52.37 cm, and 87.28 cm according to their physical distance (5 m, 10 m, 15 m, 30 m, and 50 m, respectively). In the visual matching task, the matching target was an official-size basketball (diameter 24.60 cm), and its location was controlled by a Vive control handle. The basketball was separated horizontally from the test target by 180°. The matching targets (see Fig. 1a) and test targets (see Fig. 1b–e) were placed on the grass. The fog was generated by the “viz.Scene1.fog ()” function in World Vizard 6 software. Only one parameter was applied to the function, and an exponential drop-off function was then used (OpenGL's GL_EXP); it is continuous from 0 to infinity. The concentration of fog in the VR environment was calculated by the percentage of the original scene in the foggy environment as follows: ${\text{Original~}}\;{\text{color~}}\left( {\text{\% }} \right) = {\text{~}}e^{{ - c \times d}}$ (where *c* is the concentration of fog and *d* is the distance from the origin). Because industrial pollution is common, fog is now gray rather than pure white. Therefore, the color of fog in this study was set to middle gray (0.5 0.5 0.5). The fog was placed between the participants and the test target only; no fog was placed between the participants and the matching target. Specifically, we divided the entire virtual environment into an experimental area and a matching area based on the standing position. The side of test target is the experimental area, which having fog/no-fog according to experimental design. The side of matching target is the matching area, which have no fog during the whole experiment. In order to better show this, we have drawn a top view of the whole scene (see Fig. 1f).

#### Procedures

Before the experiment, we measured each participant’s eye height, which was used to render the participant's 3D virtual environment. Participants were encouraged to look and walk around after being equipped with the HMD so that they could become accustomed to the VR scenario and equipment. We used the visual matching task to examine the observers’ egocentric distance perception, as in classical psychophysical studies^28–31^. In each trial, the participants stood on the yellow landmark. They observed the test target to judge the distance between the test target and themselves (test-target distance). After the participants finished observing and were ready to "report" the distance, they turned back and adjusted the distance between the basketball and themselves (matched distance or perceptual distance) through the gamepad until they thought it was the same as the distance between the test target and themselves. The observers were allowed to look back and forth repeatedly until they thought the matched distance was the same as the test-target distance. The adjustment process had no time limit and continued until the observers were satisfied with their matching performance. Feedback was not given in any of the trials. The experiment contained five practice trials and 60 formal trials. Twenty conditions (5 physical-distance levels × 4 fog-concentration levels, randomized) were included in the formal trials. Three trials were included in each condition. These 60 trials were entirely randomized to overcome temporal sequence and practice effects.

### Results

**Figure 2:**
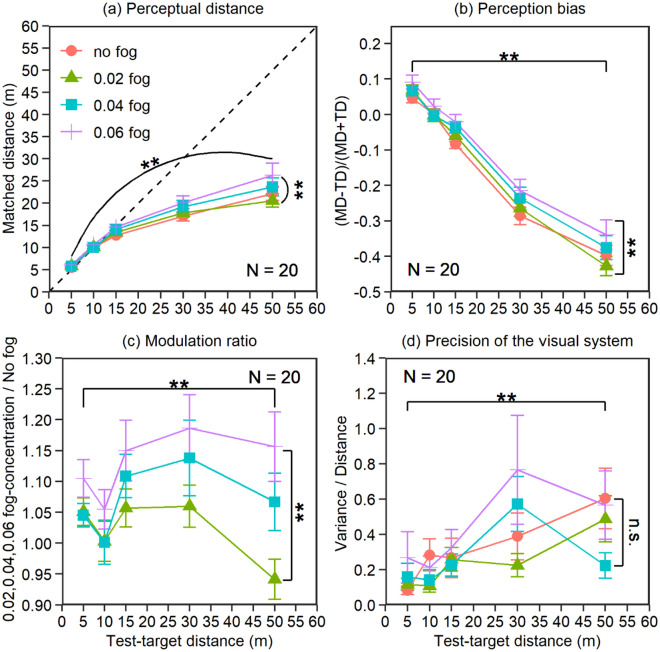
Results of experiment 1. (**a**) Original matched distance, (**b**) perception bias, (**c**) modulation ratio, and (**d**) precision of the visual system. The error bars in the figure represent the standard error.

**Figure 3:**
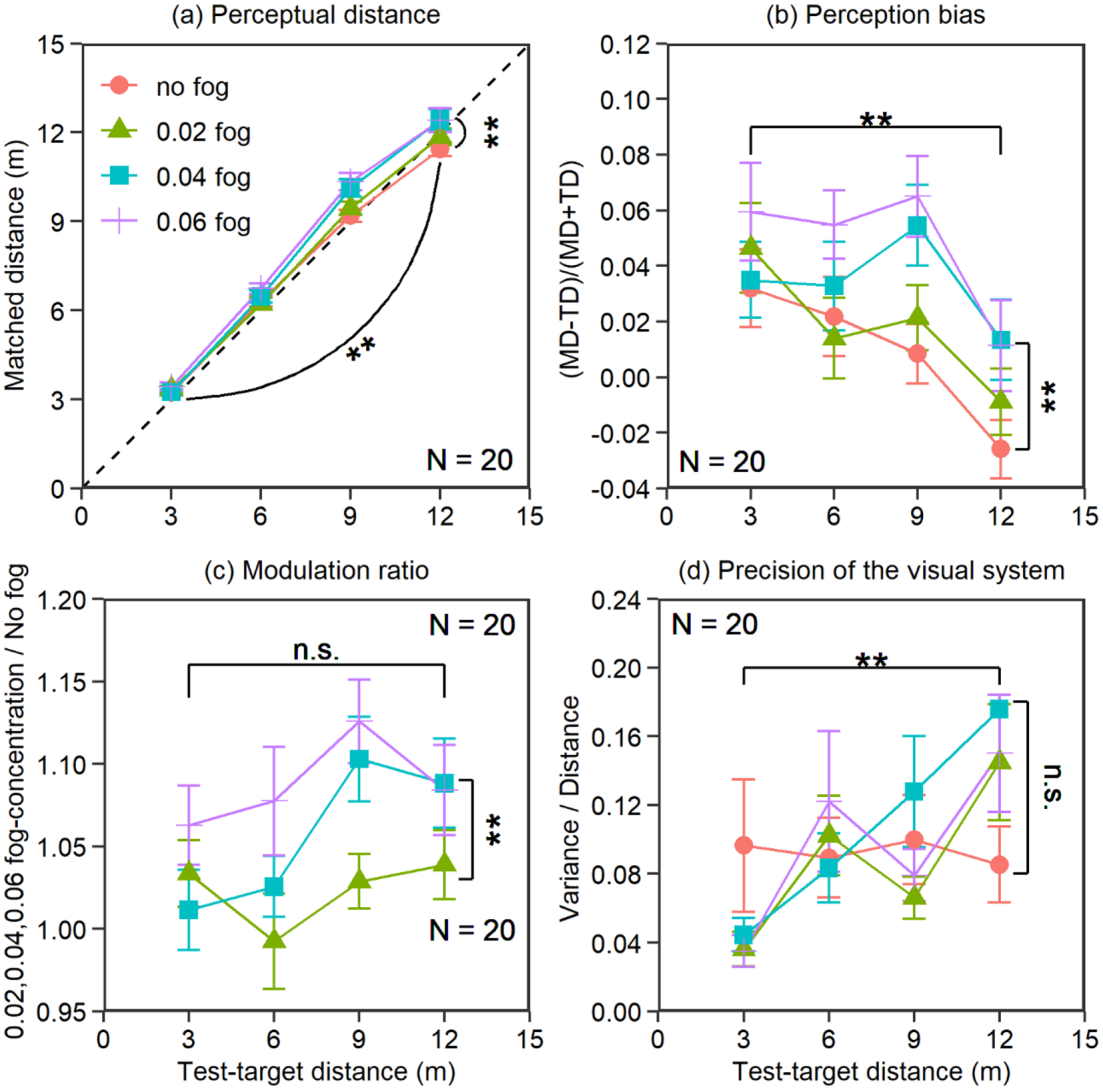
Results from experiment 2. (**a**) Original matched distance, (**b**) perception bias, (**c**) modulation ratio, and (**d**) precision of the visual system. The error bars in the figure represent the standard error.

**Figure 4:**
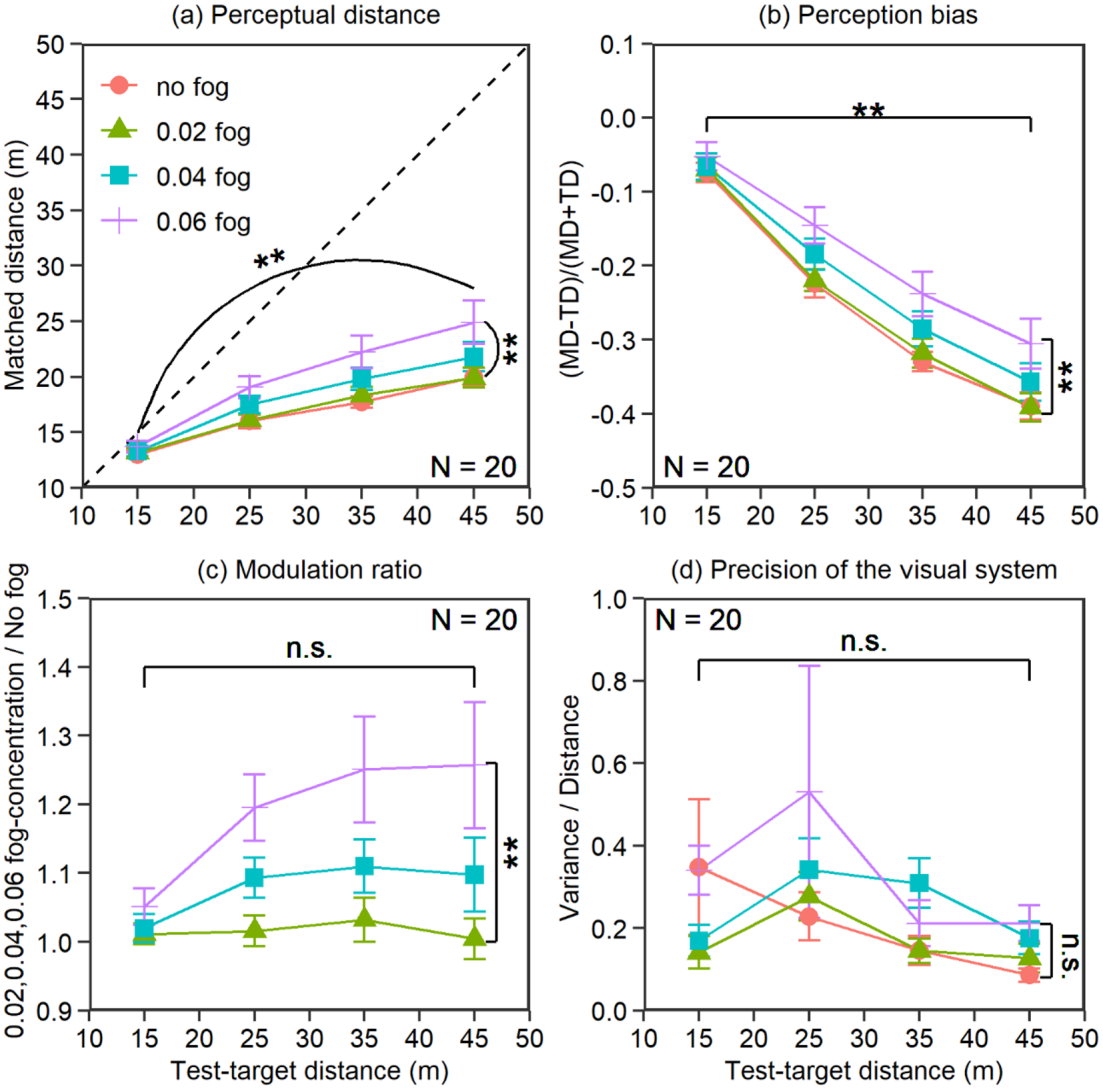
Results of experiment 3. (**a**) Original matched distance, (**b**) perception bias, (**c**) modulation ratio, and (**d**) precision of the visual system. The error bars in the figure represent the standard error.

The matched distances in the four fog-concentration conditions (no-fog/clear weather and fog concentrations of 0.02, 0.04, and 0.06) for the five test-target distances (5 m, 10 m, 15 m, 30 m, and 50 m) are shown in Fig. 2a. The dash line in the Fig. 2a. represents the situation that the matched distance and the target distance are completely equal. If the data falls in the upper left area of the dash line, it means that the participant has overestimated the egocentric distance; whereas if the data falls in the lower right area of the dash line, it indicates that the participant has underestimated the egocentric distance. The dash line in Figs. 3a and 4a has the same meaning as Fig. 2a. A two-way repeated-measures (5 test-target distances × 4 fog concentrations) ANOVA confirmed that there was a significant effect of fog concentration, *F*_1.55,29.50_ = 9.46, *p* = 0.001, Cohen’s *d* = 0.71. The positive linear trend between fog concentration and matched distance was significant (*F*_1,19_ = 10.85, *p* = 0.0038, *d* = 0.76). The higher the concentration of fog, the farther the matched distance was. Specifically, a post hoc Bonferroni analysis indicated that the matched distance in the 0.06 fog-concentration condition (MD_0.06_) was significantly greater than that in the 0.04 fog-concentration condition (MD_0.04_), *p* = 0.033; MD_0.06_ > MD_0.02_, *p* = 0.0039; MD_0.06_ > MD_no-fog_, *p* = 0.028; MD_0.04_ > MD_0.02_, *p* = 0.049; no significant difference was found between the other conditions. There was a significant effect for test-target distance, *F*_1.13,21.54_ = 75.63, *p* < 0.001,* d* = 2.00. The positive linear trend between test-target distance and matched distance was significant (*F*_1,19_ = 91.11, *p* < 0.001, *d* = 2.19). Specifically, a post hoc Bonferroni analysis indicated that the matched distance increased significantly as the test-target distance increased (MD_50_ > MD_30_ > MD_15_ > MD_10_ > MD_5_, all *ps* < 0.001). There was a significant interaction between the fog concentration and the test-target distance, *F*_3.66,69.59_ = 4.11, *p* = 0.006,* d* = 0.46. Further investigation of the interaction in Fig. 2a reveals that the relationship between MD_0.02_ and MD_no-fog_ under the test-target distance of 30 m was the opposite of that under the distance of 50 m. However, neither of them was significant (MD_30-0.02_ vs. MD_30-nofog_: *t*_19_ = 1.08, *p* = 0.30; MD_50-0.02_ vs. MD_50-nofog:_ *t*_19_ = 1.73, *p* = 0.10). Notably, when the main and interaction effects violated the assumption of sphericity, the Greenhouse–Geisser correction was used, and the degrees of freedom of the *F* test became decimals^32^. Overall, observers overestimated the egocentric distance in foggy weather compared to clear weather (i.e., the no-fog condition). Although the foggy effect was well observed and the linear trend was significant, the perceived distance did not show a perfect linear increase with increasing fog concentration. The perceived distance was significantly lower than the actual distance, i.e., the distance was underestimated when the test target was placed more than 15 m away. We speculate that this phenomenon may be because this experiment was executed in a virtual environment and was related to foggy weather, which will be discussed below.

To confirm the foggy effect, we performed two further statistical analyses. First, we normalized the matched distances (x) to the test target’s physical distance (y) by calculating the perception bias (z) with the mathematical formula z = (x − y)/(x + y). Using this formula, a z of zero indicates a perfect match; a positive z indicates overestimation, which decreases as z approaches 0; and a negative z indicates underestimation, which decreases as z approaches 0. The participants’ perception biases are shown in Fig. 2b. A two-way repeated-measures ANOVA (5 test-target distances × 4 fog concentrations) confirmed that there was a significant effect of fog concentration, *F*_1.53,29.07_ = 11.96, *p* < 0.001, *d* = 0.79. The positive linear trend between fog concentration and perception bias was significant (*F*_1,19_ = 14.51, *p* = 0.001, *d* = 0.87). The higher the concentration of fog, the larger the perception bias. Specifically, a post hoc Bonferroni analysis indicated that the perception bias in the 0.06 fog-concentration condition (PB_0.06_) was significantly larger than that in the 0.04 fog-concentration condition (PB_0.04_), *p* = 0.004; PB_0.06_ > PB_0.02_, *p* = 0.003; PB_0.06_ > PB_no-fog_, *p* = 0.006; no significant difference was found between the other conditions. There was a significant effect for test-target distance, *F*_1.47,27.99_ = 192.48, *p* < 0.001, *d* = 3.18. The negative linear trend between test-target distance and perception bias was significant (*F*_1,19_ = 234.58, *p* < 0.001, *d* = 3.51). The longer the distance was, the smaller the perception bias and the more severe the underestimation of distance. Specifically, a post hoc Bonferroni analysis indicated that the perception bias decreased significantly as the test-target distance increased (PB_50_ < PB_30_ < PB_15_ < PB_10_ < PB_5_, all *ps* < 0.001). There was a significant interaction, *F*_12,228_ = 2.10, *p* = 0.018, *d* = 0.33. Further investigation of the interaction in Fig. 2b shows that the relationship between PB_0.02_ and PB_no-fog_ under the test-target distance of 30 m was the opposite of that under the distance of 50 m. However, neither reached significance (PB_30-0.02_ vs. PB_30-nofog_: *t*_19_ = 1.40, *p* = 0.18; PB_50-0.02_ vs. PB_50-nofog_: *t*_19_ = 2.01, *p* = 0.059).

Second, we divided the matched distances in the 0.02, 0.04, and 0.06 fog-concentration conditions (i.e., foggy weather) by that of the no-fog condition (i.e., clear weather) for each test-target distance to obtain the modulation ratio (i.e., overestimation effect of foggy weather). The larger the value of the modulation ratio was, the more severe the overestimation of perceptual distance. Figure 2c shows the mean modulation ratio in all conditions. A two-way repeated-measures ANOVA (5 test-target distances × 3 fog concentrations: 0.02, 0.04 and 0.06) confirmed that there was a significant effect of fog concentration, *F*_1.46,27.70_ = 13.51, *p* < 0.001, *d* = 0.84. The positive linear trend between fog concentration and modulation ratio was significant (*F*_1,19_ = 14.51, *p* = 0.001, *d* = 0.87). The higher the concentration of fog, the larger the modulation ratio. Specifically, a post hoc Bonferroni analysis indicated that the modulation ratio in the 0.06 fog-concentration condition was significantly larger than those in the 0.04 fog-concentration condition (*p* = 0.004) and 0.02 fog-concentration condition (*p* = 0.002), which confirmed the overestimation foggy effect. The modulation ratio in the 0.04 fog-concentration condition was marginally significantly larger than that in the 0.02 fog-concentration condition (*p* = 0.056). There was no significant effect of test-target distance, *F*_4,76_ = 2.37, *p* = 0.060, *d* = 0.35. There was no significant interaction, *F*_4.10,77.97_ = 2.11, *p* = 0.086, *d* = 0.33.

To verify the stability within participants, the precision of the visual system was calculated by the variance/distance ratio (the variance in the repeated matched distance divided by the mean matched distance) in all conditions^33^. The smaller the value was, the more precise the visual system. Figure 2d shows the mean value of all observers for the five target distances in the different fog-concentration conditions. A two-way repeated-measures (5 target distances × 4 fog concentrations) ANOVA confirmed that there was no significant effect of fog concentration, *F*_1.72,32.75_ = 2.29, *p* = 0.12, *d* = 0.35, and no significant interaction, *F*_3.09,58.66_ = 1.33, *p* = 0.27, *d* = 0.26. There was a significant effect for test-target distance, *F*_2.28,43.46_ = 6.72, *p* = 0.0010, *d* = 0.59. Although the negative linear trend between test-target distance and the precision of the visual system was significant (*F*_1,19_ = 20.11, *p* < 0.001, *d* = 1.03), this difference was mainly due to the precision of the visual system at 5 m, 30 m and 50 m (5 m vs. 30 m, *p* = 0.013; 5 m vs. 50 m, *p* = 0.010). No significant effect was found between other target distances (a post hoc Bonferroni test was also used). This means that the observers’ precision of performance was constant in most conditions.

## Experiment 2

Experiment 1 demonstrated that observers overestimated the distance when the test target appeared in foggy weather. The range of test-target distances in experiment 1 covered a large scale, from 5 to 50 m, encompassing both the action and vista space. However, studies have shown that the perceptual mechanisms are markedly different in the two spaces^34^. People mainly use height in the visual field, motion perspective and binocular disparity as depth cues to perceive distance in action space, but relative size, aerial perspective, relative density, and occlusion are used in vista space. Therefore, we focus on action space and vista space in Experiment 2 and Experiment 3 respectively. In addition, as Fig. 2 shows, the perception of the subjects is more accurate within 15 m than it is beyond 15 m. Therefore, the separation point of Experiment 2 and Experiment 3 was set at 15 m. In experiment 2, we separately examined the effect of fog concentration on distance perception in action space (> 2–3 m and < 30 m) by setting test-target distances of 3 m, 6 m, 9 m, or 12 m from the observers.

### Methods

Another twenty participants (15 women, 5 men) aged 19–23 years old were recruited and were naïve to the purposes of the experiment. All of them had normal or corrected-to-normal vision and gave informed consent prior to the experiment. They received payment after the experiment. This research was approved by the local ethics committee of Suzhou University of Science and Technology and was conducted in accordance with the guidelines of the Declaration of Helsinki. The VR setup used to create the virtual scenarios and environment was the same as in experiment 1. The diameters of the four balls were 5.24 cm, 10.47 cm, 15.71 cm, and 20.95 cm according to their physical distance (3 m, 6 m, 9 m and 12 m, respectively) such that the sizes of these test balls corresponded to the 1° visual angle. The other parameters and procedures were the same as in experiment 1.

### Results

Figure 3 shows the results in the no-fog, 0.02 fog-concentration, 0.04 fog-concentration and 0.06 fog-concentration conditions for five test-target distance levels. Consistent with experiment 1, the main effect of fog concentration was significant for all three behavior indexes (perceptual distance: *F*_1.87,35.59_ = 14.98, *p* < 0.001, *d* = 0.89; perception bias: *F*_1.81,34.38_ = 14.25, *p* < 0.001, *d* = 0.87; modulation ratio: *F*_2,38_ = 20.28, *p* < 0.001, *d* = 1.03). A post hoc Bonferroni analysis indicated that the linear trend between fog concentration and the overestimation was significant for all three behavior indexes (perceptual distance: *F*_1,19_ = 23.37, *p* < 0.001, *d* = 1.11, MD_0.06_ = MD_0.04_ > MD_0.02_ = MD_no-fog_; perception bias: *F*_1,19_ = 23.00, *p* < 0.001, *d* = 1.10, PB_0.06_ = PB_0.04_ > PB _0.02_ = PB _no-fog_; modulation ratio: *F*_1,19_ = 35.63, *p* < 0.001, *d* = 1.37, MR_0.06_ > MR_0.04_ > MR_0.02_). The main effects of the test-target distance were significant for perceptual distance and bias (perceptual distance: *F*_1.47,27.87_ = 795.27, *p* < 0.001, *d* = 6.47; perception bias: *F*_1.57,29.78_ = 6.67, *p* = 0.0069, *d* = 0.59) but were not significant for modulation ratio (*F*_1.87,35.61_ = 2.72, *p* = 0.083, *d* = 0.38). A post hoc Bonferroni analysis indicated that the linear trend between test-target distance and overestimation was significant for perceptual distance and perception bias (perceptual distance: *F*_1,19_ = 983.50, *p* < 0.001, *d* = 7.20, MD_12_ > MD_9_ > MD_6_ > MD_3_; perception bias: *F*_1,19_ = 6.00, *p* = 0.024, *d* = 0.56, PB_12_ < PB_9_ = PB_6_ = PB_3_). The interaction effect between fog concentration and distance was significant for perceptual distance and bias (perceptual distance: *F*_3.89,73.85_ = 3.53,* p* = 0.012, *d* = 0.43; perception bias: *F*_4.94,93.80_ = 2.42, *p* = 0.042, *d* = 0.36) but was not significant for modulation ratio (*F*_3.63,68.90_ = 2.21, *p* = 0.083, *d* = 0.34). No significant difference was found when confirming the source of the interaction effect by paired t-test. Figure 3d shows the precision of the visual system for all observers for the five test-target distances in each condition. The main effect of test-target distance was significant, *F*_3,57_ = 6.45, *p* < 0.001, *d* = 0.58. Although the positive linear trend between the test-target distance and the precision of the visual system was significant (*F*_1,19_ = 10.85, *p* = 0.004, *d* = 0.76), only one significant trend was found by post hoc Bonferroni analysis: the precision of the visual system at 12 m was significantly better than that at 3 m (*p* = 0.013). The main effect of fog concentration and the interaction effect were not significant (fog concentration: *F*_3,57_ = 0.50, *p* = 0.68, *d* = 0.16; interaction: *F*_3.17,60.15_ = 1.20, *p* = 0.32, *d* = 0.25). This means that observers’ precision of performance was constant in most conditions.

## Experiment 3

Experiment 2 focused on the foggy effect on observers’ distance perception when the physical distance of the test target was less than 15 m. To further test the effect of fog when the distance was more than 15 m, this experiment employed 15 m, 25 m, 35 m, and 45 m physical distances to characterize the observers’ perceived egocentric distance.

### Methods

Another twenty participants (15 women, 5 men) aged 18–23 years old were recruited and were naïve to the purposes of the experiment. None of them had participated in experiment 1 or experiment 2. All of them had normal or corrected-to-normal vision and gave informed consent prior to the experiment. They received payment after the experiment. This research was approved by the local ethics committee of Suzhou University of Science and Technology and was conducted in accordance with the guidelines of the Declaration of Helsinki. The VR setup used to create the virtual scenarios and environment was the same as in experiment 1. The diameters of the four test targets were 26.18 cm, 43.64 cm, 61.09 cm, and 78.55 cm according to their physical distance (15 m, 25 m, 35 m, and 45 m, respectively) such that the size of these test balls corresponded to the 1° visual angle. The other parameters and procedure were the same as in the previous two experiments.

### Results

Figure 4 shows the results in the no-fog, 0.02 fog-concentration, 0.04 fog-concentration and 0.06 fog-concentration conditions for the five test-target distance levels. Consistent with experiment 1, the main effect of fog concentration was significant for all three behavior indexes (perceptual distance: *F*_1.26,24.02_ = 13.42, *p* < 0.001, *d* = 0.84; perception bias: *F*_1.41,26.70_ = 14.94, *p* < 0.001, *d* = 0.89; modulation ratio: *F*_1.24,23.57_ = 17.08, *p* < 0.001, *d* = 0.95). A post hoc Bonferroni analysis indicated that the linear trend between fog concentration and overestimation was significant for all three behavior indexes (perceptual distance: *F*_1,19_ = 14.00, *p* = 0.0014, *d* = 0.86, MD_0.06_ > MD_0.04_ > MD_0.02_ = MD_no-fog_; perception bias: *F*_1,19_ = 16.39, *p* < 0.001, *d* = 0.93, PB_0.06_ > PB_0.04_ > PB _0.02_ = PB _no-fog_; modulation ratio: *F*_1,19_ = 19.42, *p* < 0.001, *d* = 1.01, MR_0.06_ > MR_0.04_ > MR_0.02_). The modulation ratio scaled exactly with increasing fog concentration. The main effect of the test-target distance was significant for perceptual distance and perception bias (perceptual distance: *F*_1.42,27.01_ = 65.16, *p* < 0.001, *d* = 1.85; perception bias: *F*_1.70,32.25_ = 227.62, *p* < 0.001, *d* = 3.46) but not significant for modulation ratio (*F*_1.79,34.01_ = 2.78, *p* = 0.081, *d* = 0.38). A post hoc Bonferroni analysis indicated that the linear trend between the test-target distance and overestimation was significant for perceptual distance and perception bias (perceptual distance: *F*_1,19_ = 77.05, *p* < 0.001, *d* = 2.01, MD_45_ > MD_35_ > MD_25_ > MD_15_; perception bias: *F*_1,19_ = 390.48, *p* < 0.001, *d* = 4.04, PB_45_ < PB_35_ < PB _25_ < PB_15_). The interaction effect between the fog concentration and distance was significant for the three behavior indexes (perceptual distance: *F*_3.19,60.57_ = 3.86,* p* = 0.012, *d* = 0.45; perception bias: *F*_4.80,91.25_ = 2.59, *p* = 0.033, *d* = 0.37; modulation ratio: *F*_6,114_ = 3.22, *p* = 0.0058, *d* = 0.41). When the source of the interaction was compared by a paired t-test, the difference in performance between the no-fog and 0.06 fog-concentration conditions was not significant when targeted at 15 m (MD_15-0.06_ vs. MD_15-nofog_: *t*_19_ = 1.84, *p* = 0.81; PB_15-0.06_ vs. PB_15-nofog:_ *t*_19_ = 1.82, *p* = 0.084; MR_15-0.06_ vs. MR_15-nofog:_ *t*_19_ = 1.75, *p* = 0.096), but significant differences were revealed when targeted at 45 m (MD_45-0.06_ vs. MD_45-nofog_: *t*_19_ = 2.81, *p* = 0.011; PB_45-0.06_ vs. PB_45-nofog:_ *t*_19_ = 2.81, *p* = 0.011; MR_45-0.06_ vs. MR_15-nofog:_ *t*_19_ = 3.41, *p* = 0.03). The pattern in experiment 3 was the same as that in experiment 1. Figure 4d shows the precision of the visual system for all observers for the five test-target distances in each condition. The main effects of fog concentration and test-target distance and the interaction effect were not significant (fog concentration: *F*_1.52,28.93_ = 1.49, *p* = 0.24, *d* = 0.28; test-target distance: *F*_1.93,36.63_ = 3.27, *p* = 0.051, *d* = 0.41; interaction: *F*_2.05,38.96_ = 0.87, *p* = 0.43, *d* = 0.21), indicating that the observers’ precision of performance was constant in most conditions.

## Discussion

Our results reveal an overestimation effect of foggy weather (i.e., the foggy effect) through a visual matching task. Fog makes observers overestimate the egocentric distance, and the higher the concentration of fog, the more serious the overestimation. This modulation pattern is maintained in the action space and vista space. To our knowledge, this is the first evidence related to distance perception in foggy weather obtained with a nonverbal paradigm.

The foggy effect observed in our study is similar to that in former studies^16,19^. Observers perceive greater egocentric distance from targets in foggy weather than in clear weather. However, the degrees of overestimation measured in prior studies and this study are quite different. The overestimation effect measured by the visual matching task in foggy weather was less than 1/2 that measured by the verbal estimation task. Using a verbal estimation task, Ross (1967) suggested that the distance estimates averaged 40% higher in foggy weather than in clear weather. Specifically, the distance of a disk that was actually positioned at 9.14 m with a visual angle of 1.6 degrees was perceived to have a distance of 5.74 m in clear weather but 7.7 m (i.e., 34% greater than 5.74 m) in foggy weather^16^. Cavallo et al. (2000) used verbal estimation and found that headway overestimation averaged 25% in foggy weather^19^. In the current research, however, the largest overestimation in conditions similar to those above (i.e., < 10 m) was 12.57% (see Table 1), which was less than half that of the verbal estimation task. In contrast to the considerable overestimation observed in verbal estimation tasks in previous studies, people may still perceive egocentric distance "accurately" in foggy weather in a nonverbal paradigm. The divergence between verbal estimation and the nonverbal paradigm suggests that we may have previously overestimated the effect of fog on distance in the past.

**Table 1:** The percentage of overestimation in the three experiments (i.e., modulation ratio—1).

	Experiment 1							
	5 m	10 m	15 m	30 m	50 m			
0.02 fog	5.10%	0.30%	5.70%	5.99%	− 5.86%			
0.04 fog	4.52%	0.14%	10.87%	13.79%	6.68%			
0.06 fog	10.48%	5.48%	14.99%	18.63%	15.62%			

	Experiment 2				Experiment 3			
	3 m	6 m	9 m	12 m	15 m	25 m	35 m	45 m
0.02 fog	3.36%	− 0.75%	2.87%	3.89%	1.14%	1.58%	3.22%	0.44%
0.04 fog	1.15%	2.56%	10.29%	8.83%	1.98%	9.31%	10.99%	9.78%
0.06 fog	6.28%	7.74%	12.57%	8.41%	5.10%	19.51%	25.05%	25.70%

It can be seen that the distance perception of the target is relatively accurate within 10 m, which is possible based on binocular cues at the nearer distances. When the physical distance of the target exceeds 15 m, the foggy effect increases significantly. For example, Ross (1967) suggested that the disk that is physically positioned at 18.3 m with a visual angle of 0.7 degrees was perceived to have a distance of 10.9 m in clear weather with verbal estimation but 21.9 m in foggy weather. The distance estimates averaged 101% higher in foggy weather than in clear weather, which is twice as much as that at 9.14 m^16^. In the current research, however, the highest degree of overestimation in the visual matching task was 25.7% under the 0.06 fog concentration at 45 m (see Table 1), which is approximately 1/4 that in the verbal estimation task (i.e., 101%). These results not only confirm the advantage of the nonverbal paradigm but may also reflect the separation between action and vista space. According to Cutting & Vishton (1995), people mainly use height in the visual field, motion perspective and binocular disparity as depth cues to perceive distances in action space, but relative size, aerial perspective, relative density, and occlusion are used in vista space^34^. The stronger foggy effect beyond 15 m may suggest the influence of fog on aerial perspective.

Both the equipment (VR environment vs. real world) and the verbal/nonverbal measurements are different between this study and previous studies. Therefore, we are unable to determine whether the difference between the perceptual (matched) distance and physical distance in this study is due to the reporting method or the equipment. To avoid the effects of the equipment, we did not use “matched distance / physical distance” as an indicator of the foggy effect but adopted “the modulation ratio”. Because the modulation ratio is equal to the matched distance in the fog condition divided by the matched distance in the no-fog condition, the numerator and denominator of the formula both contain the deviations caused by the equipment, and the deviations were canceled by dividing the two. In other words, the modulation ratio is a relatively pure foggy effect that does not include the deviations caused by equipment. Indeed, like the El Greco fallacy^35–38^, although observers may misjudge the whole scale, the modulation ratio represents the relative effect of fog and comparisons based on this indicator are credible.

To eliminate the influence of retinal size on the foggy effect at different distances, we set the retinal size of all targets to the same size. This manipulation is reasonable and widely used in other studies^28–31^, but it is not perfect because it serves as a cue that the objects are all at the same distance (if the observer assumes that they are the same size, reinforced by the use of a standard-size ball). In other words, the manipulation affects the shape of the four lines (x-axis: the test target distance; y-axis: the matched distance) under each condition, but the relative location of the lines under all conditions (i.e., foggy effect) is not affected. In addition, we did not match the interpupillary distance across the participants in the experiment, which may have had some influence on perceived distance. If the presented interpupillary distance was larger (or smaller) than the observers' interpupillary distances, this would then create inaccurate binocular cues, possibly leading to a systematic overestimation (or underestimation) against the physical distance. Even this influence leads a systematic error across conditions and should not affect the experimental design, but still, future study should take this into consideration and set an appropriate interpupillary distance for each participant. Distance perception is a basic perception, and no studies have shown gender differences in distance perception, to our knowledge. Therefore, we did not consider the gender ratio when recruiting participants. However, there are some suggestions that superior spatial cognition in larger spaces for men than women, from the evolutionary psychology perspective. Extreme gender ratios in this study may have interfered with the foggy effect. Further studies should also take this into consideration.

The difference between the current research and previous experiments is not entirely due to task differences, since we did not directly compare the difference between the verbal and nonverbal paradigms in one study. We directly adopted a nonverbal paradigm, like most distance studies in the recent literature^31, 39, 40^. Other differences may also play a role. The first possible issue is the foggy weather simulation. Previous researchers used a fog chamber or conducted their experiments in actual foggy weather. In contrast, we used VR to simulate fog. The lower overestimation effect in the present study may be due to the equipment itself, specifically the borders of the HMD (110° field of view) and the imperfect simulation of the fog. The second issue may be related to the fog concentration. In previous studies, the visibility in fog was approximately 30 m, while we could use only the fog concentration settings in the VR (e.g., 0.01, 0.02, 0.06). Therefore, it is difficult to compare the concentration differences between previous studies and this study. Third, the color of the fog may have been a factor. The color of the fog in previous studies was white, while the fog color in the current research was gray. Although these differences may contribute to a reduction of the foggy effect, we believe that the current results more closely reflect the real effect of fog because studies show that the visual matching task can measure perceived distances more accurately than verbal estimation tasks^21–25,27^.

Speed perception and distance perception are the two main topics related to driving behavior in foggy weather. As described in the second paragraph of the introduction, most studies in this field have focused on speed perception^2,3,6–8,10,14,41,42^ and ultimately confirmed an overestimation effect of speed^2^. Admittedly, this overestimation effect of speed makes sense. However, while driving, drivers are less aware of their speed. They are constantly looking at the taillights of the car ahead and assessing its distance. From this perspective, distance perception in foggy weather may also be worthy of investigation.

In terms of the mechanism, an overestimation of distance can be the "starting point" for dangerous driving behavior in foggy weather. Foggy weather blurs the target, visual horizon, ground surface, and other objects in the scene. The contrast of the scene as a whole and the contrast between objects are reduced^43–46^. The contrast reduction enhances depth cues: the size of the target decreases, the angular expansion decreases^47,48^, the continuity of the ground is broken^28–30^, the sharpness of an object's edge is softened, the aerial perspective is strengthened, and stereopsis becomes more subtle^10^. As a final result, the object appears to be further away. To maintain control of the headway, the driver adopts a strategy that reduces the relative distance between his car and the car in front of him and increases his speed unconsciously. This distance-based explanation is in accordance with both the perceptual distortion hypothesis^16^ and the cybernetic perspective^20^. More interestingly, it can also be supported by the “pull and push” hypothesis^49^. From this perspective, distance perception should probably be the core concept in research about foggy weather. In the field of application, car-to-car distance control systems seem desirable to counteract biases in distance perception. This study provides systematic evidence of distance overestimation, which may provide useful parameters for designing an anti-collision system. Whereas this study focuses on distance perception under static conditions, future studies need to provide more evidence of distance perception under dynamic conditions.

In summary, we demonstrate that egocentric distance is overestimated in foggy weather through a nonverbal estimation paradigmwith a nonverbal estimation paradigm. This modulation effect is apparent in the action and vista spaces. These results reveal the internal mechanism of distance perception in foggy weather and may provide a policy basis for transportation departments.
